# The importance of applying the statement of assent to children and adolescents: a qualitative study** ***

**DOI:** 10.17533/udea.iee.v40n2e07

**Published:** 2022-09-19

**Authors:** Cristiane da Silva Varejão, Fátima H. do Espírito Santo, Maria de Nazaré de Souza Ribeiro

**Affiliations:** 1 Nurse, PhD student. National Cancer Institute. Rio de Janeiro, RJ, Brazil. Email: cristianevarejao@gmail.com Corresponding author. Instituto Nacional do Câncer National Cancer Institute Rio de Janeiro RJ Brazil cristianevarejao@gmail.com; 2 Nurse, PhD. Associate Professor, Escola de Enfermagem Aurora de Afonso Costa da Universidade Federal Fluminense. Niterói, RJ, Brazil. Email: fatahelen@hotmail.com Universidade Federal Fluminense Escola de Enfermagem Aurora de Afonso Costa Universidade Federal Fluminense Niterói RJ Brazil fatahelen@hotmail.com; 3 Nurse, PhD. Professor, Universidade do Estado do Amazonas. Manaus, Amazonas, Brazil. Email: mnribeiro2@gmail.com Universidade do Estado do Amazonas Universidade do Estado do Amazonas Manaus Amazonas Brazil mnribeiro2@gmail.com

**Keywords:** ethics committees, research consent forms, informed consent by minors, child, adolescent, comitês de ética em pesquisa, termos de consentimento, consentimento informado por menores, criança, adolescente, comités de ética en investigación, formularios de consentimiento, consentimiento informado de menores, niño, adolescente

## Abstract

**Objective.:**

To describe the importance of the Statement of Assent for children and adolescents invited to participate in a clinical study and their main reactions to its explanation.

**Methods.:**

This is an exploratory descriptive qualitative study of 17 children and adolescents, who were invited to participate in a clinical study in the field of oncology in a hospital located in Rio de Janeiro (Brazil). Data were analyzed using thematic analysis.

**Results.:**

Two thematic units were generated after data interpretation: signing the statement of assent, in which participants felt their main role when faced with the possibility of expressing their agreement or not to take part in the study; and understanding of the study, when they showed that they understood the steps of the study by asking pertinent questions to clarify their doubts. Children and adolescents understood the steps of the study contained in the Statement of Assent, were interested and asked questions to clarify their doubts about the study.

**Conclusion.:**

The Statement of Assent was important for participants understanding the study and having autonomy over their participation. As the statement strengthened the main role of children and adolescents in the research process, the conclusion was that its use in studies involving the pediatric population should be encouraged.

## Resumen

**Objetivo.:**

describir la importancia del Formulario de Consentimiento Informado para los niños y adolescentes invitados a participar en un estudio clínico aleatorizado y sus principales reacciones a su explicación**.**

**Métodos.:**

se trata de una investigación exploratoria descriptiva con abordaje cualitativo, realizada con 17 niños y adolescentes, quienes fueron invitados a participar de un estudio clínico en el área de oncología en un hospital ubicado en Río de Janeiro (Brasil). A los datos se les realizó análisis temático.

**Resultados.:**

Luego de la interpretación de los datos, se generaron 2 unidades temáticas: la firma del formulario de consentimiento y la comprensión del estudio. Los niños y adolescentes entendieron las etapas del estudio contenidas en la firma del Término de Asentimiento y se interesaron, haciendo preguntas para aclarar sus dudas sobre la investigación.

**Conclusión.:**

El consentimiento informado era importante para que los participantes entendieran la investigación y tuvieran autonomía sobre su participación. Al potenciar el protagonismo de los niños y adolescentes en el proceso de investigación, recomendamos que debe fomentarse su uso en los estudios con población pediátrica.

## Resumo

**Objetivo.:**

Descrever a importância do Termo de Assentimento para crianças e adolescentes convidados a participar de um estudo clínico e suas principais reações quanto à explicação do mesmo.

**Métodos.:**

Trata-se de uma pesquisa do tipo descritiva exploratória com abordagem qualitativa, realizada com 17 crianças e adolescentes, que foram convidados a participar de um estudo clínico na área da oncologia em um hospital localizado no Rio de Janeiro (Brasil). Os dados foram analisados empregando-se a análise temática.

**Resultados.:**

Após a interpretação dos dados, foram geradas 2 unidades temáticas: a assinatura do termo de assentimento em que os participantes se sentiram protagonistas frente a possibilidade de expressarem a concordância ou não em participar da pesquisa, e a compreensão sobre o estudo quando elas mostraram que entenderam as etapas do estudo fazendo perguntas pertinentes para esclarecer suas dúvidas. As crianças e adolescentes compreenderam as etapas do estudo que constavam no Termo de Assentimento e tiveram interesse, fazendo perguntas para esclarecer suas dúvidas com relação à pesquisa.

**Conclusão.:**

O termo de assentimento foi importante para os participantes compreenderem sobre a pesquisa e para terem autonomia sobre sua participação. Por potencializar o protagonismo de crianças e adolescentes no processo de pesquisar conclui-se que seu uso em estudos que envolvem a população pediátrica deve ser incentivado.

## Introduction

Research consists of systematic investigation using orderly methods to find answers to certain questions and solve problems. Clinical research is intended to guide professional practice for the improvement of patients’ health and quality of life. 1 Studies in the area of nursing usually emerge from problems arising during the daily practice of nurses. In Nursing, research is necessary for the development and achievement of a solid scientific basis to guarantee the quality of care provided, credibility and growth of the profession. 2 When research involves human beings, participants are required to sign the Informed Consent (IC) form. In Brazil, according to resolution 466, of December 12, 2012, of the National Health Council, the IC process involves all the steps that must be necessarily observed so that people invited to participate in a study can express themselves autonomously, consciously, freely and in an informed manner.

For children or legally incapable children, the Statement of Assent (SA) is a document in which all steps of the study are explained in accessible and appropriate language for each age group, so that they are duly clarified and can, therefore, agree or not to participate in a particular study.^3^ In this sense, studies whose participants are children and adolescents require greater attention in order to make the objectives and purposes and possible risks and benefits of the study understood. This attitude of talking to children, explaining that the procedure will only take place if they allow it, even if their responsible person has already authorized it, makes them feel respected in their right and individuality. Obtaining consent of the responsible person and the child or adolescent is fundamental for relationships in the research and a sign of respect for participants’ dignity, their ability to express opinions and their right to be heard on issues affecting them. 3

Assent, like consent, is an ongoing process that seeks to involve children in decision-making through the disclosure of key information and procedures in appropriate language, and by children’s expression of their preferences. Assent is the means by which children exercise their right to participate in the context of clinical research. It is extremely important because it includes the child in the study as a participant and establishes a relationship of trust between the child, researchers and the responsible person, thereby reducing the risks of coercion and exploitation. 4 However, studies showing the opinion of children and adolescents about their understanding and acceptance of the SA are scarce. Therefore, the aim of this article is to describe the importance of the SA for children and adolescents invited to participate in a specific randomized clinical trial and their main reactions to the explanation of the steps of the study contained in the SA.

## Methods

This is a descriptive, exploratory, qualitative study in which facts or phenomena were observed, recorded and analyzed without being manipulated.^5^ The type of design adopted allowed the description and exploration of meanings attributed by children when they were explained about the steps of a clinical study by means of information contained in the SA. This article is part of an excerpt from a data collection performed for a dissertation aimed at evaluating the effects of laser acupuncture for the relief of nausea and vomiting in pediatric patients undergoing chemotherapy. 6 It was a clinical study in which participants were randomized into two groups: one would receive true acupuncture and the other group would receive placebo acupuncture. The SA was used to explain all steps of the study and to clarify the issue of the groups (randomization) and participants’ doubts. In the greater research project of which this excerpt is part, the principles of Resolution n.466 of 2012 were followed, and it was approved by the Research Ethics Committee of the National Cancer Institute, according to opinion n. 978.441, CAAE 33745514.0.0000.5243. The data collection period was between March and November 2015.

Children and adolescents aged 6-17 years of both sexes undergoing chemotherapy for solid tumors were included in this study. The SA was prepared according to two age groups: 6-12 years and 13-17 years. This division was a requirement of the institution’s ethics committee, which felt the need for a form prepared according to each age group. Then, the statement was prepared with language and pictures aimed at each age group, for the better understanding of children and adolescents. The chemotherapy outpatient clinic where participants underwent chemotherapy was the setting. A nurse from the sector (an oncology and acupuncture specialist) applied the SA. During the explanation of the SA and clarification about the study, the behavior, reaction and speech of participants was observed. After that moment, all observations were written in a field diary.

All children and adolescents who agreed to participate in the study were invited to sign the SA. The youngest child was 6 years old, and despite not knowing how to write her name perfectly, she was encouraged and stimulated to do it her own way and within her possibilities. The Informed Consent form was given to the responsible person, who would sign and consent to the minor’s participation. For an easier understanding of the study, a language for each age group was used, as well as pictures and images to stimulate interest. Participants’ names were omitted from the study and each one was instructed to choose a codename to protect their identities. Each codename presented was chosen by children and/or adolescents themselves. They were explained that even with authorization of their legal guardians, they would need to assent and sign the document, authorizing their participation in the study, and they were not obliged to participate if they did not want to.

Data were analyzed following the steps of thematic analysis. 7 For the operationalization of this process, after transcribing the dialogues, the material was read for the exploration of contents, and treatment and interpretation of results obtained. Speeches were classified using a colorimetric method, that is, words and expressions with the same meaning were grouped in the same color and codes were generated, giving rise to two thematic units: signing the assent term, and understanding about the study. In order to avoid limiting the analysis to the researcher’s view, two more researchers worked independently in the formation of codes and thematic units. Afterwards, there was a meeting between researchers for discussion and consensus in relation to discrepancies.

## Results

Seventeen participants were interviewed, including children and adolescents; nine were male, eight participants were aged 6-12 years, and nine were 13-17 years old. (Table 1).

**Table 1:** Characterization of participants

Codename	Age	Sex
Guerreiro de Jeová	6	M
Ana	9	F
Menguinho	10	M
Pokemon	10	M
Anita	10	F
Elza	11	F
Mulher Maravilha	12	F
Batman	12	M
Bem	13	M
Margarida	13	F
Peter Pan	13	M
Rosinha	14	F
Rapunzel	14	F
Dragon Ball Z	15	M
Cinderela	16	F
Junior	16	M
Neymar	17	M

### Signing the statement of assent

The approach to participants aged 6-12 years old, as they were younger, occurred through a game, always with the responsible person next to them, so they felt safer. They showed interest and were receptive from the first moment of the approach. They wanted to be sure that their responsible person would be there with them at the time of conversation, as shown in the statements: *Can my Mom hear it too?* (Elsa); *Mom ... stay here and hear this with me* (Mulher Maravilha).

It was made clear that their permission and signature was essential and that even if their responsible person authorized it, they would not be included in the study if they did not want to. Although indispensable, parental consent alone was not sufficient. Children have guaranteed rights and the right to a voice. Thus, it is essential that the researcher guarantees conditions for the child’s choice of participating or not in a certain study. During the dialogue, it becomes clear how crucial the responsible person’s consent is for children’s participation in the study: *Mom, I’m going to sign now, can I? Have you already signed?* (Menguinho); *Mom, we both have to sign, okay?* (Elsa); *Mom, sign it too, along with me* (Pokemon);

*Mom! Sign yours too!* (Batman).

Only one out of the eight children aged 6-12 years old (Guerreiro de Jeová, 6 years old) took the SA to be signed at home. When he returned with the signature, the mother reported that upon arriving home, the child sat at his little table very happy to sign, demonstrating satisfaction for deciding on the participation in the study. Like him, other children showed satisfaction in signing the term: *See, mom, I’m important... I had to sign it...* (Guerreiro de Jeová); *See mom, I also have to sign. Just like you* (Anita).

At the first moment, when participants aged 13-17 years were asked if they could be approached to talk about the study, they nodded their heads to demonstrate a yes, consenting to the approach. They were usually wearing headphones or lying with their entire bodies covered. They did not ask questions and when asked if they had understood the explanation about the SA, they simply responded with gestures (nodding or shaking the head to deny). Only one out of the nine participants in this age group signed the SA after explanation about the study. Eight took the form home and brought it signed in the next meeting. Adolescents were more reticent in the first contact, and had to take the SA form to be read at home and clarified their doubts in the next meeting. The following speeches demonstrate this attitude: *Can we talk later? It’s just that I’m sleepy*... (Ben); *Can you talk later?* (Margarida); *Do I have to sign today? Ah... I’d like to think... Can I answer later?* (Neymar); *I’m not signing today, okay?* (Junior); *I’ll take it home...* (Dragon Ball Z).

### Understanding about the study

Participants in both age groups asked questions about the study, wanting to understand the research better. However, in the younger age group, these questions were asked on the same day of the explanation about the study. And the most frequent doubts were about pain, especially about pain in laser acupuncture: *Will it be painful and how will I know if the research worked or not?* (Batman); *Does the laser hurt? How long does it take to do the laser?* (Anita); *Will I feel pain?* (Guerreiro de Jeová); *Will it hurt, nurse?* (Pokémon); *What will I feel?* (Mulher Maravilha).

In the age group of 13-17 years, doubts were clarified in a second meeting, and the issue of pain was not so much questioned. Their most frequent questions were about the steps of the study. Teenagers’ speeches demonstrate the desire for clarifications. In the second meeting, an adolescent declared not feeling comfortable participating in the study, understanding that it would be the place of a guinea pig: *I don’t want to be a guinea pig... I don’t want to be used for testing* (Junior). After this report, it was explained that the laser had already been used with children and adolescents in other studies and the researcher just wanted to see its effect in relieving post-CT nausea and vomiting. After this new conversation, the teenager understood the matter and signed the SA, demonstrating comprehension of this step of the study.

Like Junior, other adolescents also expressed their doubts about the intervention that would be performed. None of them was left without the proper clarification: *Is laser acupuncture as good as needle acupuncture?* (Margarida); *How will I know if it worked or not?* (Cinderela); *Can I leave the study later, if I want to?* (Neymar). The study in question was about the application of laser acupuncture that would always be performed immediately before chemotherapy. On the day of the procedure, when a nurse from the Chemotherapy Center (CT) went to see Anita to administer chemotherapy, the patient said: *No Miss, you have to wait. The research nurse said that the light* (word she used to identify the laser) *has to be done before chemotherapy* (Anita).

The chemotherapy nurse explained that she would wait and reported the child’s speech to the researcher. This shows how much Anita understood the procedure and was attentive to the steps of the study protocol. There was a withdrawal from the study. A few days after signing the term, Ana said she no longer wanted to take part in the study: *I don’t want to do the laser anymore... I want to stop doing it, but my Mom wants me to continue...* (Ana). The researcher talked to the mother and explained that the daughter’s wish had to be respected. She also asked Ana if that was what she wanted and the child reaffirmed her desire. It was clarified that nothing she did not allow would be done and she was free to withdraw from the study. At that moment, Ana became calmer and thanked her.

In the clinical study for which patients were being invited to participate, there was randomization into two groups: one group with true laser acupuncture and the other with placebo laser acupuncture. A lot of care was necessary to make the understanding of this issue of division of groups easier, and the explanation was repeated as many times as needed. Still, some children did not understand the existence of two groups: *But why do you have to have two groups?* (Dragon Ball Z); *I want to be into the real group, because I feel sick a lot* (Rosinha); *Why can’t I know which group I’m in, if in the true or false group?* (Rapunzel).

In view of the above, a new explanation was necessary and very calmly, the researcher explained the randomization process and its importance. She made it clear that she would not be the one to choose; children would do it themselves by picking the envelope in which there would be a letter directing them to one of the groups.

## Discussion

In this study, where the importance of applying the statement of assent was evaluated, it was found that participants had doubts to be clarified. Through assent, it is assumed that underage participants should be helped in a way that is appropriate to their level of development, so they understand the nature of the study and have all their doubts clarified.^8^ As in the case of this article, Lambert and Glacken,^9^ in a study to obtain the signature of children and adolescents for a survey, also formulated statements of assent for two age groups; one for the group of children aged 6-10 years and the other for children aged 11-16 years. The authors reinforce the importance of attention and care with the size of the letter used, the choice for simple words without technical terms, and an attractive and interactive design. They also emphasize the scarcity of studies on the subject and the importance of disseminating experiences of signing statements of assent and informed consent forms by children and the responsible person. 9

In the present study, it was very important for pediatric patients to decide if they accepted or not to participate in a clinical study. However, for the range of participants aged 6-12 years, the agreement of the responsible person was fundamental and made them more confident and certain of the decision. In a study conducted with children to identify their knowledge about the SA, 47.8% confirmed that someone could influence their decision to participate in the investigation; most cited the mother (69.1%), followed by the father (13.2%), and the others mentioned some other type of person, such as another family member or the health professional. 10 This result is in agreement with what was observed in the present study regarding the age group of 6-12 years. However, for participants aged 13-17 years, at first, the consent of the responsible person did not prove to be decisive for these teenagers’ participation in the study. Autonomy and the right to decision-making must be guaranteed to participants. Together with their responsible person, they must be involved in decision-making about their participation or not in research or treatments. 11,12

The ability to use information and weigh risks and benefits regarding different options to make a choice was evaluated in a review that demonstrates the emergence of this skill in late childhood and early adolescence, that is, from the age of 12 or 13 years.^13^ During this period, children begin to reason abstractly about certain situations, alternatives and consequences, to combine multiple variables in a more complex way and examine information in a systematic and exhaustive way. These results converge with those of the present study, where children’s doubts were mainly about the issue of pain. The older ones (13-17 years old) had questions about randomization, the effect of laser acupuncture and what would happen if they wanted to withdraw from the study. These questions demonstrate a greater ability to understand information, generating more complex doubts about the procedures. 13

Participants must be given the right to withdraw from the study whenever they wish, without compromising their treatment and care. The participant Ana had her desire respected when she wanted to leave the study. Participants in a study must be aware they are engaging in a voluntary activity that can be stopped without personal consequences, 14 and their autonomy and decision must be considered, as declared in the SA they signed. Children need to feel comfortable and fearless to talk to the researcher about their wishes regarding participation or not in the study. 14

As for approach strategies for children in research situations, their peculiarities, developmental needs and individual characteristics must be taken into account. Therefore, a SA was designed for each age group taking part in the interview. It is important that the researcher knows the way of thinking, feeling and acting at different ages. Therefore, each stage of contact with the child must be planned, although modifications and adaptations may be necessary during interactions. 14,15 The adolescents interviewed in the present study (with the exception of one) did not sign the SA on the same day they were approached. They received the document and a recommendation to read it at home and talk with their responsible person or with whom they trusted. In this context, Lind et al. 15 report that an individualized view of adolescents and their limits brings them closer to the researcher and increases their engagement with the study, providing the necessary tools for their understanding.

Later, when adolescents returned with their doubts, they seemed more receptive to clarifications. In this sense, researchers must show understanding and interest in children/adolescents and above all, make an effort to convey their real intention, clarifying their doubts so that they understand and consequently, can decide whether or not to participate in the study.^8^,^12^ In a survey that estimated the opinion of parents and children (minors) in studies on informed consent, the general view of adolescents was that they should have control over any decision involving them.^16^ Such data corroborate the present article, where adolescents were not concerned with the signature and consent of their responsible persons.

Every participant needs to understand why they are invited to participate and what the study is like. The components included in the statement of assent aimed at the pediatric population must differ and be formulated according to the level of development of each age group.^17^ In addition, during the approach to children, researchers must assess their understanding, including the use of other methods and strategies, such as drawing, photography and stories to make themselves understood.^18^ Children need to be informed according to their understanding and their participation must be well planned; they are the ones who teach us the path and the way it should be followed.^19^ Therefore, with this research it was possible to give voice to pediatric participants. The importance of a SA formulated for each stage of development and explained according to children’s ability to understand was observed. This was found by considering that they understood the steps of the study, clarified their doubts and had the option of signing or not, thereby deciding on their participation in the study.

## Conclusion

 Obtaining the SA signature in studies of children and adolescents requires tools aimed at the age groups of participants. Although the informed consent form must be signed by the responsible person because participants are underage, the authorization of children or adolescents involved must also be obtained, thus considering their ability to understand and make decisions.

In this study, the act of deciding whether or not to sign the SA was important for participants. Children aged 6-12 years signed the SA on the same day, and approval by the responsible person was essential so they could feel safe and agree to participate in the study. On the other hand, participants aged 13-17 years (with the exception of one) signed the SA in a second meeting and did not show much concern regarding the consent of the responsible person. Both groups understood the steps of the study, expressed their fears and had their doubts answered.

By strengthening the protagonist role of children and adolescents in the research process, we concluded that the use of the SA in studies involving the pediatric population should be encouraged. More studies related to the topic should be conducted, so the participation of the pediatric population in research is done ethically and conscientiously.
